# Demography of reintroduced Mount Kenya guereza (*Colobus guereza kikuyuensis*) at Karura Forest, Kenya

**DOI:** 10.5194/pb-12-1-2025

**Published:** 2025-02-18

**Authors:** Peter Fundi, Shadrack Muvui Muya, Winnie Kiiru, Robert Nesta Kagali

**Affiliations:** 1 Department of Zoology, Jomo Kenyatta University of Agriculture and Technology, 62000 – 00200 Nairobi, Kenya; 2 Department of Environmental Studies and Resources Development, Chuka University, 109 – 60400 Chuka, Kenya; 3 Mpala Research Centre, 555 – 10400 Nanyuki, Kenya

## Abstract

Between May 2014 and March 2016, 22 groups of Mount Kenya guerezas (*Colobus guereza kikuyuensis* Lönnberg, 1912) were reintroduced in Karura Forest, Kenya. To assess the success of the reintroduction, we conducted monthly censuses over 8 years (2016–2023). We determined group size and composition of the reintroduced population. During the censuses, we recorded instances of births, deaths (disappearance), dispersal from natal groups, and the habitat types where groups established home ranges. A total of 14 of the reintroduced groups settled along the riverine zones. Over the years, we recorded an annual increase in the number of births and group size, indicating successful adaptation of the reintroduced primates. Coming from a degraded source habitat, the primary cause of death was related to arboreality adaptation (23 %), and only one case of infanticide during a group takeover was recorded. Group fusion was not observed; however, seven groups with more than two adult males began splitting in 2019. By December 2023, 109 births had been recorded, and groups increased to 31 due to natal dispersal. Our results demonstrate that *C. guereza kikuyuensis* reintroduction to Karura Forest was a success.

## 1 Introduction

Several successful animal translocations have been undertaken for varying reasons and with different goals and means while achieving varied success rates (Fischer and Lindenmayer, 2000; Soorae, 2011; Strum, 2005; Richard-Hansen et al., 2000). The success of such translocations is determined by pre-translocation planning involving the assessment of the species' habitat requirements and the search for and selection of a suitable habitat (Chauvenet et al., 2013; Kleiman, 1989). The most obvious criterion suggested for determining post-translocation success is the establishment of a self-sustaining population (Griffith et al., 1989; Franquesa-Soler et al., 2022). Monitoring the demographic development of translocated animals is a must to assess the success of the translocation (Goossens et al., 2005; King et al., 2012; Batson et al., 2015).

The initial success of reintroduction can be assessed by measuring post-release survival and reproduction (King et al., 2012), including social environments, leading to groups' settlement (Berger-Tal and Saltz, 2014). Monitoring can be done either by regular censuses of a given population or through long-term monitoring of known individuals and groups (Pusey et al., 2007). However, post-release monitoring of translocated wildlife poses several challenges (Dias et al., 2023), including the inability to follow groups and funding constraints (Berger-Tal et al., 2020). Additionally, obtaining accurate demographic data for long-lived species can be difficult (Robbins and Robbins, 2004). The problem is even worse in reintroduced populations where sample sizes are usually low (Nichols and Armstrong, 2012).

**Figure 1:**
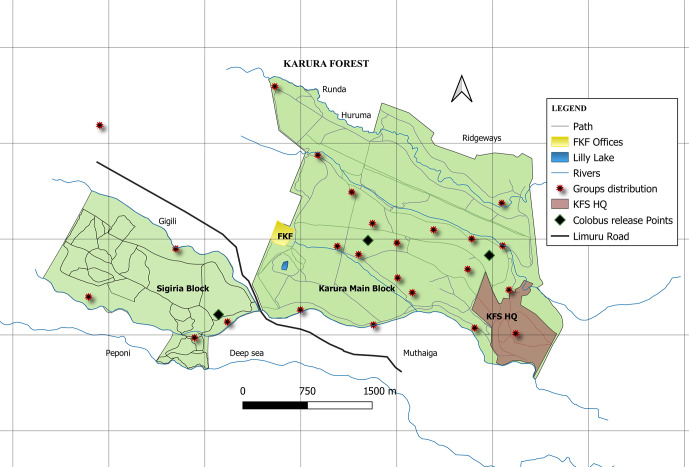
A map of the Karura Forest reserve showing the two forest blocks where *C. guereza kikuyuensis* groups were released (diamond black points denote release points) and post-release groups' settlement distribution (red stars show the centres of group's locations recorded during monthly censuses).

Between May 2014 and March 2016, 22 Mount Kenya guereza groups were translocated from the fragmented and highly degraded riverine ecosystems in Nyandarua County to Karura Forest (Fig. 1), a distance of around 130 km. The mean group size was 8 ± 2 individuals and the total number of translocated individuals was 142 (34 adult males, 50 adult females, 23 juvenile males, 25 juvenile females, and 10 infants). The subspecies is believed to have historically occupied Karura Forest, as evidenced by the presence of a wild *C. guereza kikuyuensis* group located approximately 2 km from the forest. To assess the success of the reintroduction, we monitored the survival of the translocated primates, as well as changes in group size and composition between 2016 and 2023. Here we report the demographic development of the re-introduced population.

## 2 Methods

### 2.1 Ethical statement

No animal handling was involved in this study; therefore, ethical consent was not necessary. Permission to conduct the study was obtained from the Department of Zoology at Jomo Kenyatta University of Agriculture and Technology and the Friends of Karura Forest, a Community Forest Association (CFA).

### 2.2 Study site and study species

Karura Forest is an upland urban forest located 4 km north of the city of Nairobi, between latitudes of 1°14^′^26^′′^ to 1°14^′^33^′′^ S and longitudes of 36°47^′^44^′′^ to 36°50^′^38^′′^ E, with its highest point at 1800 m above sea level. The forest was established as a reserve in 1932 and is jointly managed by the Kenya Forest Service (KFS) and the Friends of Karura Forest (FKF). The average annual rainfall is 930 mm, with a maximum of 1240 mm during the long rains from April to June and a minimum of 350 mm during the short rains between October and December. Temperatures within the forest vary throughout the year according to the season, cloud cover, and sunshine.

The forest comprises stands of natural forest and plantation forest and sections with a mix of both natural and exotic trees. Consequently, the plant community includes both natural trees and forest plantations. The natural forest has an extensive canopy cover averaging 85 % and hosts about 20 mammalian species and over 110 bird species. The forest is a major tourist hub within Nairobi, attracting both local and international visitors. An urban road (Limuru Road) divides the forest into two blocks (Sigiria and Karura), which are physiognomically distinct. Sigiria is largely a mixed forest of both natural and exotic trees, while Karura consists of continuous stands of natural and plantation forests.

The guerezas were released into the Karura Forest reserve between May 2014 and March 2016 after each group was kept in a holding cage for 3 nights prior to release for acclimatization. The groups were released at three sites, one in Sigiria (4 groups) and two points in Karura main block (18 groups). The groups were named in alphabetical order from letter C to X in order of release. Post-release monitoring of the population was initiated immediately after each group had been released into the forest. Only two groups were habituated to the researchers' presence. All groups were, however, recognizable based on their size, composition, and home range position, while coat colour and facial and tail features were used to differentiate between individuals in a group.

### 2.3 Data collection

A cross-sectional research design was used to assess changes within groups and the whole population. The design involved conducting monthly censuses of all the groups to determine changes in group size and composition. A group was defined using the “chain rule” (Ramos-Fernández, 2005) of 50 m, where all individuals seen within 50 m of another individual were considered part of the same group. A few groups having distant neighbours would however spread beyond 50 m and still be treated as one group. We stayed with each group for 20 min, took the GPS location of the group, counted individuals, and recorded their age and sex classes. We classified juveniles and subadults as juveniles. Additionally, we counted newborn infants (white coat colour of the infant) to infer birth and also counted all deaths. We examined dead individuals to determine the possible cause of death, and when necessary, haematological tests were conducted at the Kenya Institute of Primate Research.

We categorized the habitat where we found the groups into five types (riverine, open canopy, closed canopy, mixed forest, and plantation forest). Information on group spread within their settlement site was recorded. This was computed as the mean of two diameters measured across the group in different orientations. Instances of individual's natal dispersal were also recorded. We regarded the case when an individual(s) departed from its group of birth (Teichroeb et al., 2011) for more than 2 months.

### 2.4 Analysis

The data were tested for homogeneity of variance using Levene's test, and the Shapiro–Wilk test was used to test for normality. To test the influence of social organization on group dispersal distance after release, Student's t test was used to examine differences in group settlement distance in relation to the release point for uni-male (8 groups) and multi-male (11 groups) groups, as well as for groups with fewer than six ( N = 10) and more than six ( N = 12) individuals. To examine released group dynamics after translocation, we compared group size, births, and mortality of each group. We compared group sizes 6 months and 8 years after release using a paired t test.

Using the years (2016–2023) as the predictor and mean number of individuals as the response variable, one-way ANOVA was conducted to compare annual variation in group size, and a Tukey honest significant difference (HSD) test was conducted when a significant difference was found among group means. The Kruskal–Wallis test was used to test for annual differences in group sizes when the data did not meet the normality test. In this test, we used the year (2016–2023) as the predictor variable and group size as the response variable. Regression analysis was used to examine whether differences in social organization could contribute to change in population growth rate by examining the relationship between the number of adult females in a group and group sizes with the number of births over the 8-year period. Additionally, the coefficient of determination ( $R^{2}$ ) was used to analyse the change in mortality over the years. Owing to the few instances of natal dispersal, only group size was considered as the dependent variable associated with group split. A paired Student's t test was used to examine the influence of group size and sex composition during splitting.

## 3 Results

### 3.1 Descriptive results

The mean number of males (including juvenile males) translocated per group was 2.55, and the mean number of females (including juvenile females) was 3.32, and their mean difference was not significant ( Z = 1.52, df = 21, p = 0.13). During release, 36.6 % of the groups ( N = 8 groups) had one adult male, 36.6 % ( N = 8 groups) had two adult males, 13.3 % ( N = 3 groups) had more than three adult males, and 13.3 % ( N = 3 groups) had females only.

### 3.2 Groups' settlement

Upon release, 63.6 % of the groups ( N = 14) settled along the Karura, Getathuru, Thigiri, and Turaco rivers (Fig. 1) at distances between 10–140 m from the riverine. A two-sample Student's t test indicated a significant difference in the mean distance from the river between groups settled farther from the river ( > 150 m) and those along the river ( t = 4.97, df = 1, p < 0.05). The difference in group settlement distance from the holding cage was not significant ( t = 0.48, df = 9, p = 0.64) for groups with ≤ 1 male ( $\bar{x}$ = 1037.1 m, SEM = 255.22 m) and groups with ≥ 2 males ( $\bar{x}$ = 1185.3 m, SEM = 254.45 m). Additionally, the difference in settlement distance for groups with ≤ 6 individuals ( $\bar{x}$ = 1250.3 m, SEM = 283.4) and groups with > 6 individuals ( $\bar{x}$ = 1039.5 m, SEM = 174.19 m) was not significant ( t = 0.63, df = 10, p = 0.54). Two bachelor groups, each comprising two males, settled in areas outside Karura Forest, one at City Park (2.8 km from the release point) and the second one at Red Hill Estate (2.7 km from the release point). The group spread (the distance between the two furthest individuals in a group) ranged from 0 to 60 m (mean 18.6 m ± SE 0.42).

### 3.3 Population monitoring

Data on annual group size between March 2016 (when all 22 groups were released) and December 2023 were tested for normality using the Shapiro–Wilk Test and did not deviate from normal distribution ( p > 0.05). Soon after release, population growth declined, with mean group size data for the first 6 months ( $\bar{x}$ group size at release = 6.24 and $\bar{x}$ group size after 6 months = 5.32) indicating a significant difference using a two-sample Student's t test ( t = 3.13, df = 21, p < 0.01). Comparing the difference in group size between release and 8 years later ( $\bar{x}$ group size in December 2023 = 9.51), the difference was significant ( t = 5.77, df = 21, p < 0.01).

The census data indicated that the population has been growing steadily since the release of the first group in 2014. Levene's test was conducted to assess the variance in population size year after year, indicating the variances were not homogenous ( p = 0.04). Comparing the mean number of individuals across the years using one-way ANOVA indicated a significant difference ( F = 3.06, df = 9, p < 0.01). Tukey's HSD test indicated a significant difference in group sizes between the years 2016 and 2023, between 2017 and 2023, and between 2017 and 2023 ( p < 0.05).

### 3.4 Growth rate

#### 3.4.1 Births

**Figure 2:**
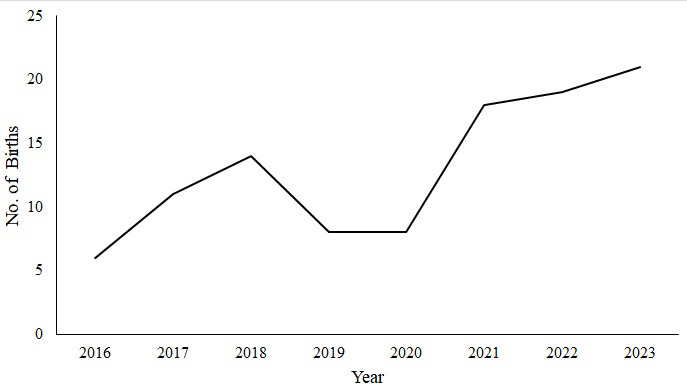
Trend in number of births for the whole population between 2016 and 2023.

The number of births has been increasing annually; however, these numbers declined in 2019/2020 due to a prolonged drought (Fig. 2). The data on the number of births over the 8-year period deviated from a normal distribution (Shapiro–Wilk test, p < 0.05), and the Kruskal–Wallis test did not indicate a statistically significant trend ( H = 10.45, df = 7, p = 0.16).

The relationship between the number of females in a group and the number of births was moderate ( R = 0.57, F(1,20) = 9.72, p < 0.05) and positively correlated. The relationship between group size and the number of births over the 8-year period was, however, strong ( R = 0.71, F(1,20) = 19.77, p < 0.05) and also positively correlated.

#### 3.4.2 Mortality

Over the 8-year period, a total of 26 deaths were recorded, with the numbers varying annually and zero deaths recorded in 2020. The number of deaths has been increasing over time, though at a slow rate ( $R^{2}$ = 0.33). The main causes of colobus monkey deaths were tree falls, where individuals snapped their necks ( N = 6); electrocution by uncoated electricity overheads ( N = 3); diseases ( N = 2); razor wire from neighbouring residential houses ( N = 2); and infanticide ( N = 1). The cause of death for a large percentage (46 %, N = 12) of the recorded cases was unknown.

### 3.5 Natal dispersal

**Figure 3:**
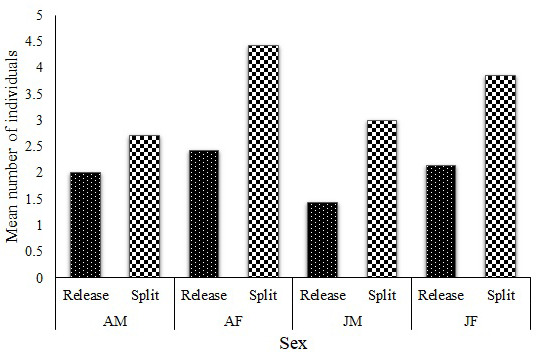
Mean group composition at release and at the point of splitting (AM, adult males; AF, adult females; JM, juvenile males; and JF, juvenile females).

During the entire period, the colobus groups were followed (since the release of the first group), and no single case of group(s) or individuals merging (fusion) was recorded. One group (group H), consisting of three adult males (AMs), three adult females (AFs), and three juvenile males (JMs), split into three smaller groups (H_1_ = 1 AM, 3 AF and 1 JM; H_2_ = 1 AM, 1 JM and H_3_ = 1 AM, 1 JM) immediately after release. Seven other groups started splitting in 2019, and the comparison of group size between release ( M = 8.4, SD = 1.4) and the time of dispersal ( M = 14, SD = 2.6) was significant ( t = 7.41, df = 6, p < 0.01). A graphical presentation of the group composition data at release and at the point of split shows an increase in all the age–sex classes (Fig. 3), with a significant difference in male ( t = 5.2, df = 6, p = 0.01) and female ( t = 7.84, df = 6, p < 0.01) compositions.

Apart from group H, which split into three smaller groups, the seven other groups split into two groups. In these cases, only one adult male left the natal group with a few females and juveniles. The situation was different for group W, which had four males at the time of the split; two males left with a few females and juveniles. In all split cases, infants and their mothers remained in the natal group. Despite groups U, W, and X splitting in almost equal numbers (6 : 7, 7 : 6, and 7 : 6 respectively), the mean group sizes of the split and original groups were significantly different ( t = 2.97, df = 6, p = 0.02).

## 4 Discussion

More than half of *C. guereza kikuyuensis* groups released at Karura Forest were within the normal group size range of 3–15 individuals, consisting of one to two adult males, two to four adult females, and their offspring (Chapman and Pavelka, 2005). However, a few groups had more than two males ( N = 3), while others were composed of females only ( N = 3). The abnormal composition could have resulted from social grouping within the highly degraded source site, a condition observed in group H which split upon release. A similar observation had been reported in chimpanzees (Lehmann et al., 2007).

While the released colobus groups show a strong preference for settling near rivers, factors such as the number of males or the overall group size do not significantly affect the distance they settle from the release point. A similar post-release habitat selection scenario has been reported in black howler monkeys in Belize where all the study groups concentrated their activities in riverine zones (Ostro et al., 2000). Riverine forests are associated with succulent young leaves, tall trees, and continuous canopy, making it ideal for colobus monkeys, which largely depend on the moisture content of their diet (Kim, 2002).

Released bachelor groups tend to disperse further from the release point, as indicated by two of the three groups that split from group H. These two bachelor groups moved out of the release forest block, with one group settling in the nearby City Park forest and the other settling within the community area that still has natural tree cover. Compared to mixed groups, colobus bachelor groups may have a tendency to explore and settle further away from the release point. However, the released groups maintained typical within-group spatial dispersion, with some variability depending on habitat type.

The reintroduced *C. guereza kikuyuensis* population has demonstrated resilience and growth despite an initial decline in group size shortly after release. The early decline in mean group size could have been due to various stressors and challenges faced while adapting to the new environment. No births were recorded in any of the groups 6 months post-release. This could be associated with elevated stress levels during translocation, which may disrupt hormonal balance and affect the breeding cycle (Dickens et al., 2010). However, over the 8-year period, the mean group size and the total number of individuals significantly increased, indicating a steady annual rise. These results suggest a positive trend in the population's adaptation and growth. Despite the annual increase in number of recorded births, there was a drastic decline in births in 2019/2020, coinciding with the longest drought experienced during the study period. Similar reproductive inhibition was recorded in spider monkeys during prolonged drought periods (Campos et al., 2020).

A few small groups recorded zero births over the 8-year monitoring period despite having females in the group. Group D had two individuals, a male and a female; group P had two males and one female; and group F had a male and a female. None of these groups settled in any defined territory. This indicates that factors other than just the presence of females and group size might be influencing reproductive success. However, the strong positive correlation between group size and number of births underscores the importance of group dynamics and social interactions in reproductive success.

Over the 8-year period, few deaths were recorded relative to births, though a high number of deaths occurred 6 months post-release. The leading cause of death was tree falls, where individuals snapped their necks as they adapted to arboreal life. Some individuals also explored fenced areas outside the forest dying from injuries. Despite being arboreal, the source habitat was greatly degraded, and the drastic change in habitat upon release into the Karura Forest posed initial adaptation difficulties (Letty et al., 2007). The cause of death for a large percentage of recorded mortalities was unknown, as individuals were discovered a few days after dying, with some already decomposing. Only one case of infanticide was recorded after an attempted male takeover, and the infant had a canine bite on its skull.

Fusion was not observed during the 8-year period colobus monkeys were monitored. In cases where individuals dispersed from natal group, split individuals formed separate groups, rather than temporary subgroups typical of many nonhuman primates displaying fission–fusion behaviour (Spider monkeys, Aguilar-Melo et al., 2018; Chimpanzees, Lehmann et al., 2007; Yunnan sub-nosed monkeys (Xiang and Grueter, 2007). The ecological constraints model links group size in social animals to seasonal food availability (Teichroeb and Sicotte, 2009). In reintroduced *C. guereza kikuyuensis*, group split did not follow any particular seasonal pattern, indicating that food availability was not a limiting factor for group size. This pattern is common among folivorous species where leaves are abundant and evenly distributed relative to fruits (Janson and Goldsmith, 1995). The reintroduced population exhibited a distinct pattern of natal dispersal, driven by increases in group size and changes in sex composition. Groups with two or more adult males split, with one male departing with a few females. In chimpanzees and spider monkeys, subgroup sizes were reduced with a reduction in food resources (Chapman et al., 1995). This natural process helps manage group sizes and ensures social stability within the population.

Finally, the analysis of long-term post-release monitoring data illustrates that the reintroduction of *C. guereza kikuyuensis* to Karura Forest was successful in terms of post-release reproduction. The initial goal of establishing a self-sustaining colobus population within the forest seems to have been attained, owing to the high number of births compared to the deaths. Further studies are however recommended to generate more quantitative demographic data and facilitate the development of a model to predict the probability of population persistence, providing an indication of long-term reintroduction success.

## Data Availability

The data that support the findings of this study are available from the corresponding author upon request.
